# Nonconventional Ca(OH)_2_ Treatment of Bamboo for the Reinforcement of Cement Composites

**DOI:** 10.3390/ma13081892

**Published:** 2020-04-17

**Authors:** Luz Adriana Sanchez-Echeverri, Jorge Alberto Medina-Perilla, Eshmaiel Ganjian

**Affiliations:** 1 Departament of Mechanical Engineering, CIPP-CIPEM Research Group, Universidad de los Andes, Bogotá 111711, Colombia; jmedina@uniandes.edu.co; 2 Facultad de Ciencias Naturales y Matemáticas, Universidad de Ibagué, Carrera 22 Calle 67, Ibagué 730002, Colombia; 3 School of Energy, Construction and Environment, Built & Natural Environment Research Centre, Coventry University, Priory Street, Coventry CV15FB, UK; cbx111@coventry.ac.uk

**Keywords:** alkali treatment, bamboo fibers, cement composites, flexural strength, physical properties, biomass for reinforcement

## Abstract

This study compares the structural and morphological changes in *Guadua angustifolia* Kunth (GAK) fiber prepared in three different ways (chips, barkless and crushed) when non-conventional alkaline treatment is applied. Moreover, it shows the improvement of mechanical properties of cement composites reinforced with these treated fibers. The three different preparations of *Guadua* were treated with a saturated solution of calcium hydroxide (5%) at 125 °C and 1.25 kPa for 3 h to remove non-cellulosic compounds. Then, their chemical, morphological, and structural properties were examined. The fibers exhibiting the higher delignification rate were selected to prepare cement composite boards, whose mechanical properties were successively compared with those of composites reinforced with untreated *G. angustifolia* fibers. The water/cement ratios of the cement mixed with the Ca(OH)_2_-treated and the untreated fibers were, respectively, around 0.3 and 0.25. The flexural strength and toughness of the two composites were evaluated after 7, 28, and 90 days of curing. The calcium hydroxide treatment showed higher efficiency in removing non-cellulosic materials when performed on crushed bamboo; moreover, the mechanical properties of the composites reinforced with the treated fibers were higher than those mixed with the untreated ones. After 90 days of curing, the flexural strength increased by around 40% and the toughness became three times higher (*p* < 0.05). The mechanical improvement by the Ca(OH)_2_ treatment of *G. angustifolia* fibers demonstrates its potential for the fabrication of cement composites.

## 1. Introduction

In recent years, there has been a fast rise in the use of renewable natural fibers as reinforcing agents of cement materials [1,2] in order to reduce their environmental impact. Non-wood materials are attracting growing attention due to their easy availability, low cost, rapid renewability, and eco-friendly nature [3,4,5,6]. Bamboo plants are one of the most promising sources for such materials, especially because of their abundance and rapid growth as well as their long fibers, which have superior strength properties compared with others [7,8]. *G. angustifolia* Kunth is a native bamboo of South America. It is widely utilized as a construction material due to its high mechanical resistance; however, only a part of its culm is used and the potential applications of its other parts are yet to be fully studied. Many researchers have tried to reinforce cementitious materials with bamboo fibers since they can remarkably improve the fracture toughness and flexural strength of cement composite boards (CCBs) while simultaneously reducing their total weight [9,10,11]. These improvements depend mainly on the source and morphology of the fibers, the interaction at the fiber–matrix interface, the manufacturing technology, and the fiber pretreatment. All the natural fibers, including bamboo fibers, have an intrinsic hydrophilic nature, which is a drawback for a reinforcement agent since they become swollen, weakening the mechanical properties of the composite material. Thus, several chemical modifications have been tested to increase the fiber roughness and the amount of cellulose exposed on their surface [12,13,14].

In this viewpoint, alkali treatments are one of the major techniques adopted to obtain fibers with adequate properties for reinforcement applications due to their low cost. They can change the morphology of natural fibers by removing the impurities from their surface; for example, they can disrupt the lignin structure and break the linkage between lignin with the other carbohydrate components of lignocellulosic biomass [15]. These pretreatments provide bamboo fibers with an acceptable delignification degree as well as high yield and viscosity. Bamboo lignins generally have more phenolic hydroxyl groups than other biomass [16]. Biomass pulping is an alkali treatment method widely used in the paper industry; it is usually performed via a chemical process called Kraft pupling [17], which uses sodium hydroxide and sodium sulfide. The high pulp strength, the efficient improvement of the fiber adhesion to the cement matrix, and the ability to handle almost all species of softwood and hardwood give the Kraft process advantage over other pulping processes. However, it also has some drawbacks; for example, it requires a high pulping temperature and produces sulfur-containing volatile compounds [18].

Therefore, other alkali treatments have been considered for the production of pulp fibers with adequate properties. Among them, calcium hydroxide treatment is a useful method for selectively reducing the lignin content of lignocellulosic biomass without a significant carbohydrate loss [19,20]. Moreover, some researchers have defined it as a great option based on its cost, operation, health hazard, sustainability, and recoverability; Ca(OH)_2_ is the least expensive per kilogram of hydroxide treatments [21]. Despite these benefits, this technique has not been fully studied as an alternative fiber pretreatment for the reinforcement of cementitious materials.

In this work, part of the GAK culm was used to prepare three different samples: chips, barkless, and crushed. These samples were treated with a calcium hydroxide solution to obtain adequate fibers to be used as reinforcement agents for cementitious materials. The treated fibers were characterized via Fourier-transform infrared spectroscopy (FTIR), X-ray diffractometry (XRD), and scanning electron microscopy (SEM). Furthermore, the effect on the flexural strength and toughness of CCBs was studied.

## 2. Materials and Methods

### 2.1. Materials

**Figure 1:**
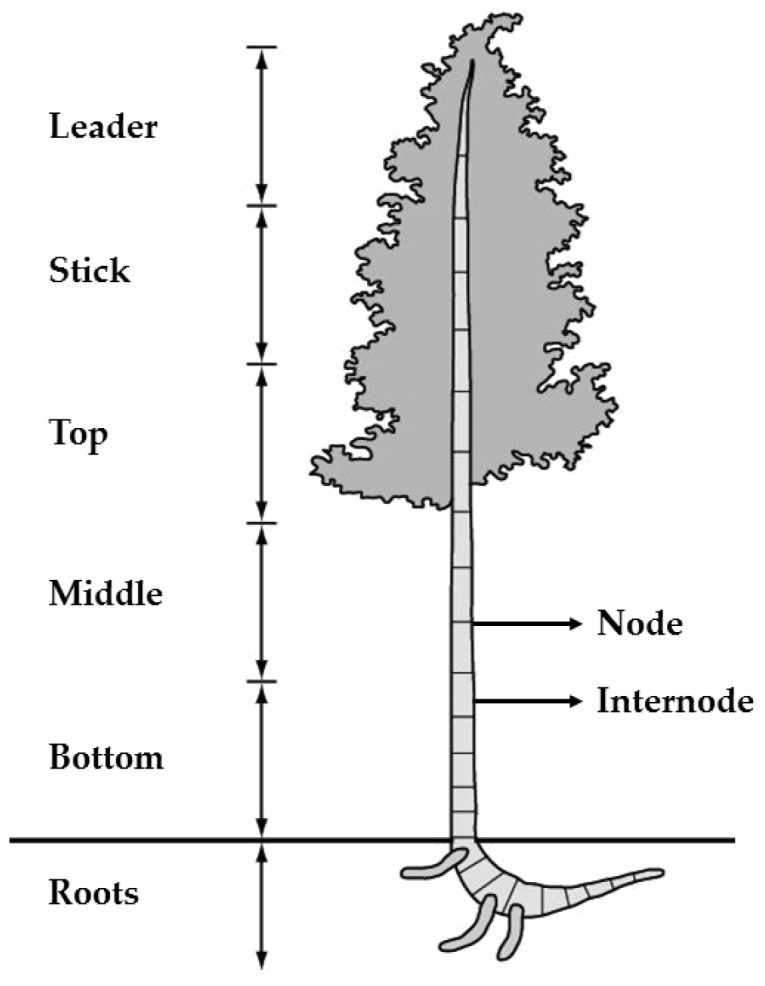
Parts of the culm of *Guadua angustifolia* Kunth.

GAK is a fast-growing bamboo plant with a straight and hollow cylindrical culm, which is divided into different parts throughout its height (Figure 1).

GAK samples were taken from the internodes of the middle culm, which is the part most used for construction in Colombia and is widely commercialized, of plants grown for four years in an experimental field in La Tebaida, Quindío (central region of Colombia).

Ordinary Portland cement type I supplied by a local company was used to produce cement composites boards, the specifications of cement are summarized in Table 1.

### 2.2. Sample Preparation

**Figure 2:**
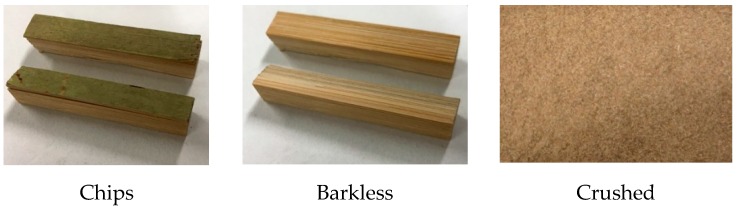
*G. Angustifolia* Kunth culm samples, prior to the calcium hydroxide treatment.

GAK internodes were cut on a band saw machine (Shop Fox W1706, Bellingham, WA, USA) obtaining chips with approximate dimensions of (60 mm × 10 mm × 15 mm). To understand the influence of the Ca(OH)_2_ treatment on the sample preparation, three different sample shapes were prepared from the chips obtained, and named as follows (Figure 2):

- Chips: the culm internodes were cut into small samples (60 mm × 10 mm × 15 mm);
- Barkless: the barks and internal layers of the chips were removed with a scalpel;
- Crushed: the chips were crushed in a blade mill and sieved with a 16 mesh (1.18 mm).

### 2.3. Ca(OH)_2_ Treatment

**Figure 3:**
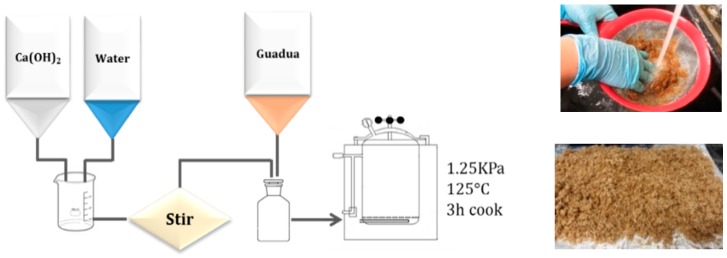
Ca(OH)_2_ treatment process for the *G. angustifolia* samples.

100 g of dried samples were immersed in solutions of 5% of calcium hydroxide (Panreac^®^) with a liquor/sample ratio of 10:1. The solutions were cooked in an autoclave for 3 h (1 h to reach the cooking temperature of 125 °C and 2 h of cooking at 1.25 kPa). Then, the samples were washed with tap water to remove the excess Ca(OH)_2_ solution and, finally, were air-dried for 48 h at room temperature (Figure 3).

### 2.4. Fiber Surface Characterization

Scanning electronic Microscope SEM (JEOL JSM-6490LV, JEOL, Peabody, MA, USA) was used to examine the effect of the Ca(OH)_2_ treatment on the surface of the GAK samples. The samples were cut into small pieces and attached on a carbon adhesive after being coated with a gold film to make them conductive. The observation was conducted in the low-vacuum mode at a 15 kV electron acceleration voltage and 12–20 Pa; images of different surfaces were obtained with the backscattering electron signals.

### 2.5. Fourier Transform Infrared Spectroscopy (FTIR)

The FTIR technique was utilized to analyze the changes in the chemical structure of the GAK after the Ca(OH)_2_ treatment. A Thermo Electron Corporation spectrometer (Nicolet 380 FT-IR, Thermo Electron Scientific Instruments LLC, Madison, WI, USA) and standard KBr pellets were used. The samples were chopped, passed through a #70 (200 µm) sieve, mixed with 300 g of KBr, and, finally, pressed into pellets (diameter of 16 mm). Five scans were performed for each sample between 400 and 4000 cm^−1^; the resulting spectra were analyzed with the OMNIC software (Thermo Electron Scientific Instruments LLC, Madison, WI, USA).

### 2.6. X-Ray Difraction

The influence of the Ca(OH)_2_ treatment on the crystallinity of the GAK fibers was investigated with XRD. The diffraction patterns of the treated and untreated samples were recorded in the range of 4°–70° with 2θ range with a Rigaku Ultima III diffractometer (Rigaku, Tokyo, Japan) operated at 40 KV, 40 mA, and room temperature, with Cu Kα radiation (wavelength of 1.5406 Å) and a step size of 0.05 s. The Jade 9.0 software (9, KS Analytical Systemd, Aubery, TX, USA) was used for analysis. The crystallinity index (CI) was calculated according to the Segal empirical method [22] as follows (Equation (1))

(1) $$CI\left(\%\right)=100\;\left[\frac{\left(I_{002}-I_{am}\right)}{I_{002}}\right],$$

where I_002_ is the maximum intensity of the (0 0 2) crystalline peak at 22°–23° and I_am_ is the minimum intensity of the peak at 18–19° [12].

### 2.7. CCB Manufacturing

After the Ca(OH)_2_ treatment, the sample that shows the higher variation in chemical composition according to the analyses of XRD and FTIR, was chosen as the reinforcement agent for the successive preparation of cementitious materials. The pretreated fibers (5% dry weight) were mixed with water (450 mL) and cement (130 g). The CCBs were prepared in the laboratory via a slurry vacuum de-watering technique; they were manufactured in a slab shape with a 200 mm length and a 100 mm width for the flexural test. To form and compact the boards, a 120 KN load was applied for 10 min by a compression testing machine (Avery Denison 3000, Denison Mayes Group, Hunslet, Leeds, UK). The CCB thickness was measured before the flexural test. Once the boards were formed, they were demolded and cured at 95% humidity and 25 °C. For comparison, other CCBs were similarly produced by using untreated fibers; boards without any reinforcement agent were also fabricated as a control. The thickness of CCB was about 5 to 6 mm.

### 2.8. Water Cement Ratio of CCBs

The water/cement ratio (W/C) influences the mechanical properties of cement boards. This ratio is commonly calculated as a mass of added water divided by the cement mass; however, in the slurry method, the vacuum removes excess water, whose final water in the CCB is, therefore, different from the initial one. In this study, it was estimated after the demolding process based on the weight difference. The mass of the water retained after the manufacturing process was calculated as follows (Equation (2)):

$$w_{H_{2}O\;loss}=w_{total\;mix}-w_{final},$$

(2) $$w_{H_{2}O\;retained}=450\;ml-w_{H_{2}O\;loss},$$

where $w_{H_{2}O\;loss}$ is the mass of water lost during the manufacturing process, $w_{total\;mix}$ is the mass of all components (water, cement, and fibers) used in the mixture, and $w_{final}$ is the weight of the demolded CCB.

### 2.9. Flexural Strength of CCBs

A three-point loading flexural test with a 200 mm span and 5 mm/min velocity was performed according to BS EN 12467:2012 with a JJ Lloyd tensile testing machine (AMETEK Test & Calibration Instruments, Bognor Regis, Sussex, UK). To evaluate the reinforcement effect, the test was conducted on samples prepared with calcium hydroxide treated fibers, untreated fibers, and without fibers. Five samples were tested after 7, 28, and 90 days of curing (Category D); their thickness was measured at four different points and the average value was used to derive the modulus of rupture (MOR).

### 2.10. Toughness of CCBs

The toughness was evaluated by calculating the specific energy (SE) after 7, 14, and 28 days of curing through the Equation (3) [23].

(3) $$SE=\frac{Absorbed\;energy}{be},$$

where Absorbed energy is the integration of the stress-deflection curve between the beginning of the elastic behavior and the reduction in carrying the capacity to the maximum stress [24], and *b* and *e* are the sample width and thickness, respectively.

To compare the results on W/C, flexural strength and toughness between different samples, an analysis of variance (ANOVA) was carried out; the intervals were calculated to determine whether there were significant differences. Then, the Tukey test that is a single-step multiple comparison statistic was performed by SPSS^®^ software (25, IBM Corp. ©, Armonk, NY, USA) to locate these differences.

### 2.11. Fracture Surface of CCBs

SEM microscope JEOL JSM-6490LV (JEOL, Peabody, MA, USA) operated in the low-vacuum mode was used to examine the CCB fractured surface after the flexural test. The samples were cut in small pieces and attached on a carbon adhesive after being covered with a gold film to make them conductive. The analysis conditions were similar to those described in Section 2.4.

## 3. Results and Discussion

### 3.1. Ca(OH)_2_ Treatment Effect on the Bamboo Fiber Surface Morphology

**Figure 4:**
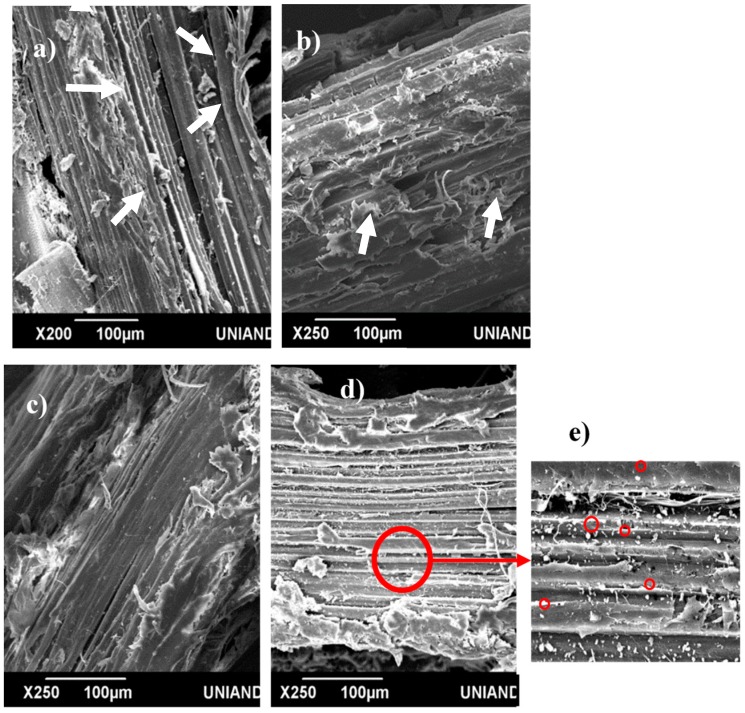
Scanning electron microscopy images of (**a**) untreated and (**b**–**e**) Ca(OH)_2_-treated *G. angustifolia* samples: (**b**) chips, (**c**) barkless, and (**d**,**e**) crushed (the circles in (**e**) indicates the calcium compounds on the surface).

**Figure 5:**
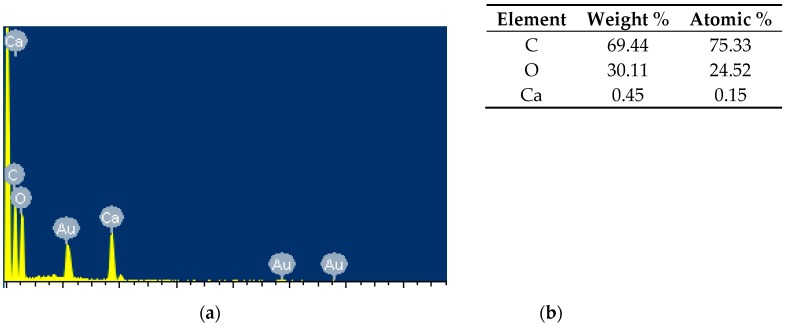
(**a**) EDS Image of nodules observed over the crushed sample surface, (**b**) quantity of elements spread on the deposited nodules in the surface showed in Figure 4e.

Figure 4 compares the surface of the various Ca(OH)_2_-treated GAK samples with that of untreated. The figure shows that impurities such as waxes and oils (arrows) present in untreated samples have been removed after Ca(OH)_2_ treatment from the surface of the barkless and crushed samples, but it did not produce any notable modification in the chips. Over the crushed sample also showed a deposit of nodules on the surface (Figure 4e); these nodules can be associated with calcium compounds [19]; which were confirmed with EDS analysis (Figure 5).

**Figure 6:**
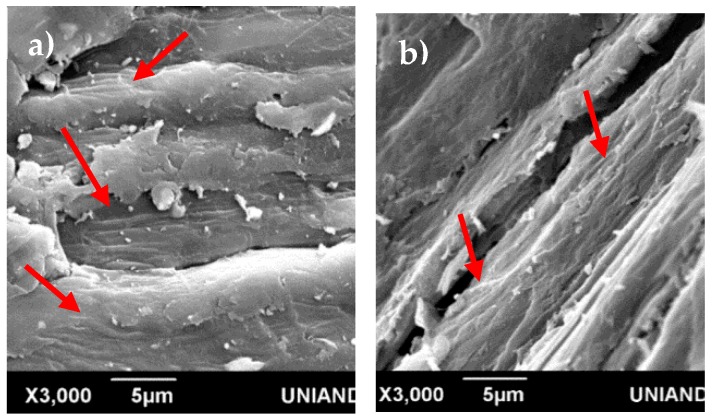
Scanning electron microscopy images of the fiber surface of the (**a**) barkless and (**b**) crushed samples after Ca(OH)_2_ treatment.

Figure 6 shows in more detail the surface for the barkless and crushed samples after Ca(OH)_2_ treatment; the red arrows indicate the elementary fibrils, which were exposed by the efficient treatment with more effectiveness on the crushed surface. Binding materials such as pectin, lignins, and hemicelluloses were probably removed due to the easy penetration of the calcium ions, inducing fiber fibrillation.

### 3.2. FTIR Analysis of Untreated and Ca(OH)_2_-Treated Bamboo Fibers

**Figure 7:**
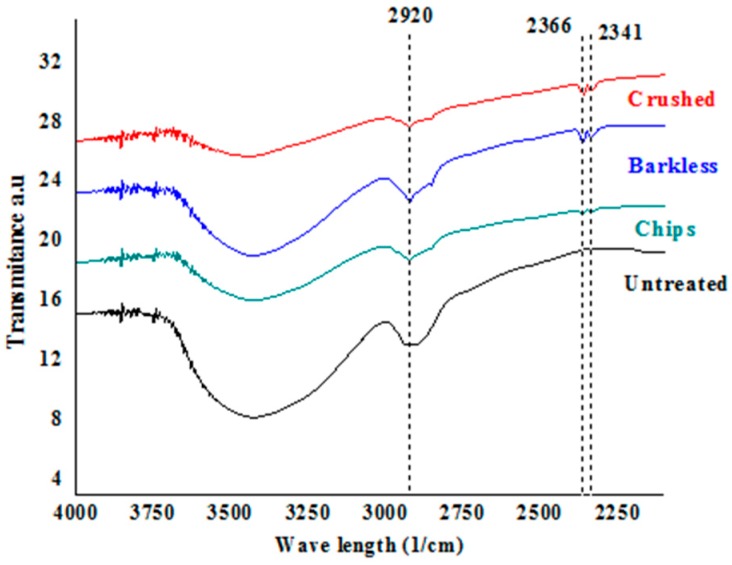
Fourier-transform infrared spectra in the 4000–2100 cm^−1^ range of untreated and Ca(OH)_2_-treated *G. angustifolia* samples.

**Figure 8:**
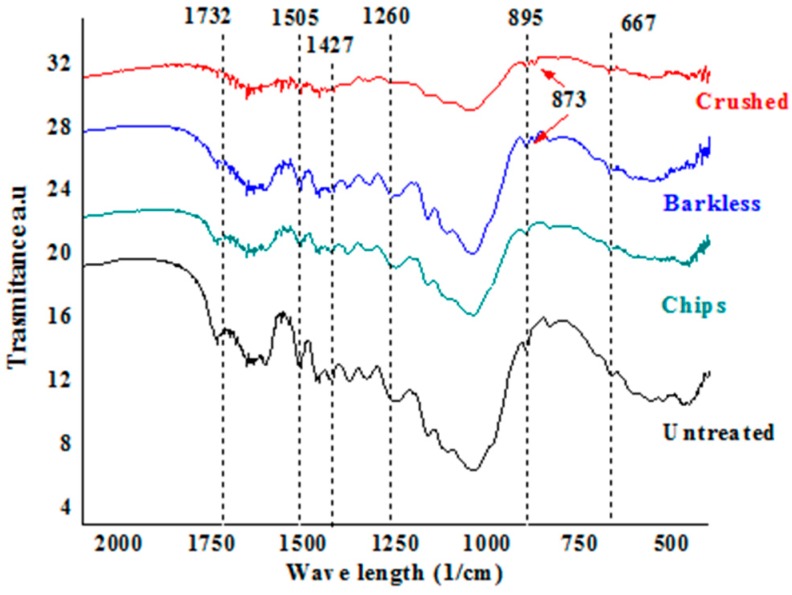
Fourier-transform infrared spectra in the 2100–500 cm^−1^ range of untreated and Ca(OH)_2_-treated *G. angustifolia* samples.

Figure 7 and Figure 8 display the FTIR spectra of the samples in the 4000–2100 cm^−1^ and 2000–400 cm^−1^ wavenumber ranges, respectively. The band related to the presence of waxes and oils (2920 cm^−1^) was significantly reduced in all samples after the alkali treatment (Figure 6), which had cleaned the surface; this reduction was more noticeable on the crushed sample. The vibration band of gaseous carbon dioxide, observed at around 2366 and 2341 cm^−1^, was related to the background CO_2_ in the spectrometer; although the background was subtracted in all the spectra, those of the crushed and barkless samples still exhibited this signal due to their higher accessibility to calcium ions.

Another visible differences before and after the Ca(OH)_2_ treatment was the modification in the band at 1732 cm^−1^ (Figure 8), which is associated with the stretching of CO groups in hemicelluloses; this band almost disappeared in the crushed sample and was weaker in the barkless sample in contrast with the chips one. The signals at 1505 and 1427 cm^−1^, respectively related to lignin [25] and C=O vibration in carbonate ions, also decreased after the treatment. This indicates that the alkali treatment removed the non-cellulosic compounds, which is consistent with previous reports [26,27]; however, this removal was not fully effective in the studied samples since their bands could still be observed. The reduction of non-cellulosic compounds facilitates the mechanical separation of the fibers, which is beneficial for their use as reinforcement agents.

The spectra of the crushed and barkless samples also showed a band at 873 cm^−1^. Gunasekaran and Anbalagan have attributed this signal to calcite [28], which could be formed as a protective layer by the reaction between the carbon dioxide resulting from the carbohydrate degradation and calcium hydroxide, preventing further carbohydrate degradation [20].

### 3.3. XRD Analysis of Untreated and Ca(OH)_2_-Treated Bamboo Fibers

**Figure 9:**
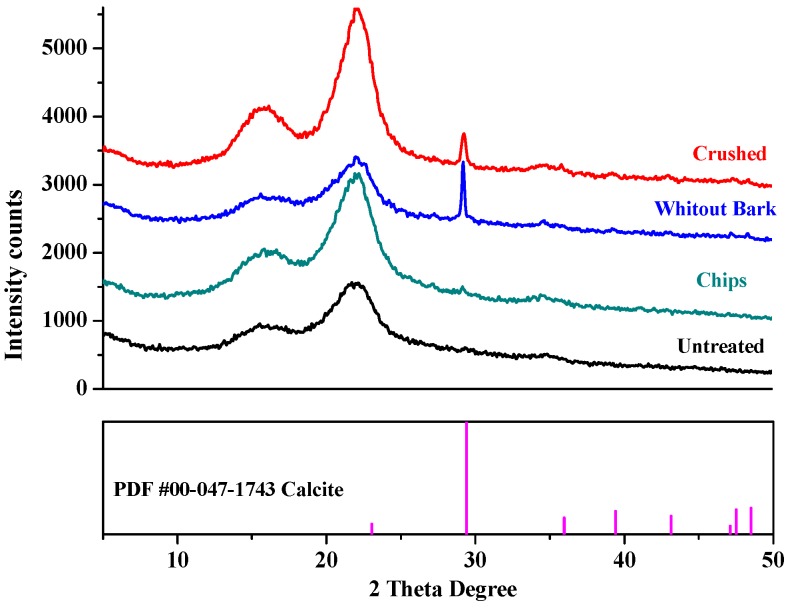
X-ray diffraction patterns of the untreated and Ca(OH)_2_-treated *G. angustifolia* samples, along with PDF #00-047-1743 calcite.

Figure 9 displays the XRD patterns of the various samples investigated, along with the powder diffraction file of calcite (PDF#00-047-1743) taken from the organic material database of the Jade 9.0 software. The untreated sample exhibited a sharp, high peak at 22.7° and two overlapped, weaker peaks at 15° and 16.3°, attributed to cellulose I with crystallites having the preferred orientation along the fiber axis [14,29]. All the Ca(OH)_2_-treated samples showed diffraction patterns similar to that of the untreated one. However, in the spectra of crushed and barkless, samples an additional peak appeared at around 29°, which was assigned to calcite, whose presence was suggested also by the FTIR results.

Alkali treatments increase the *CI* of vegetable fibers, which is related to the arrangement of the cellulosic chains. Table 2 summarizes the crystallinity indexes derived from the XRD patterns. Alkali treatments hydrolyze the amorphous parts of the cellulose in fibers, increasing the crystalline cellulose portion [30,31,32] which explains the *CI* increase in the samples after the Ca(OH)_2_-treatment. This effect was consistent with to the removal of the amorphous constituents of the fibers, such as lignins, hemicelluloses, pectin, and amorphous cellulose, as reported also by other authors [26,33].

However, the essential peaks of the GAK fibers were not shifted after the treatment, which indicates that there was no transition from cellulose I into cellulose II and the relative carbohydrates were not significantly altered [14,34]. Therefore, the *CI* variations originated only from the elimination of amorphous compounds such as lignins and hemicelluloses.

### 3.4. W/C of CCBs

Based on the results of fibers after alkaline treatment, crushed samples were selected to manufacture due to the efficiency of calcium treatment in this sample.

The W/C influences the strength of a composite. W/C is generally defined as the ratio between the initial cement and water weights used for the mixture [35]. With the same W/C, the addition of steel fibers significantly improves the compressive strength of concrete after 28 days [36]. However, in the slurry method, the vacuum process removes excess water, whose final water in the CCB is, therefore, different from the initial one. Table 3 lists the average W/C values measured for CCBs reinforced with Ca(OH)_2_-treated and untreated fibers and for those without fibers, indicating that the fiber inclusion increased the W/C with more intensity in the treated calcium fibers. It was coherent with the water retention capacity of the fibers. The Tukey test (*p* < 0.05) showed a significant difference on the W/C ratio for all CCB.

### 3.5. Mechanical Properties of CCBs

**Figure 10:**
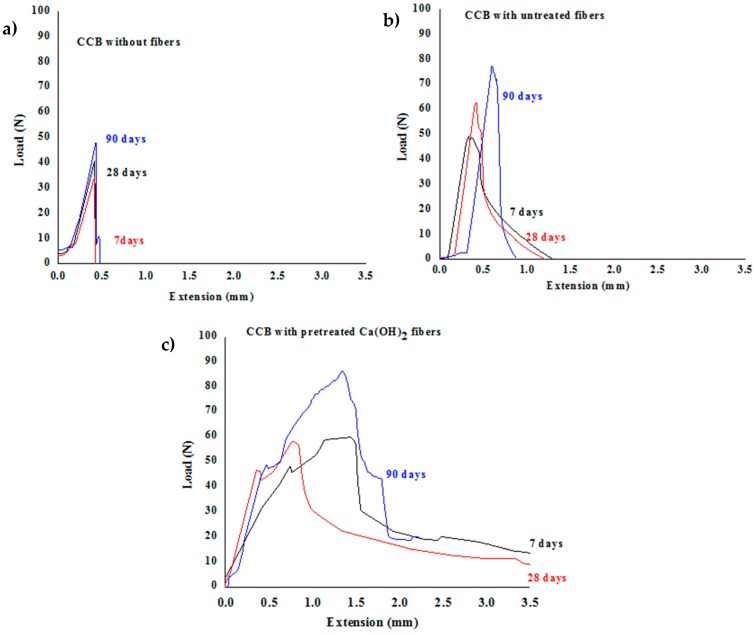
Load–extension curve of various composite cement boards (CCBs) after 7, 28, and 90 days of curing (**a**) CCB without fibers, (**b**) CCB with untreated fibers, and (**c**) CCB with calcium treated fibers.

Figure 10 shows the load-extension curves of the flexural strength test of different composites. Table 4 summarizes the MOR and SE of the various CCBs after 7, 28, and 90 days of curing. In all the cases, the MOR increased along with the curing time, while the SE did not significantly change.

The use of GAK fibers improved the flexural strengths of the CCBs. The highest variation with curing time was exhibited by the CCB reinforced with untreated fibers, which increased from 3.6 to 7 days up to 7.4 MPa and over 100% after 90 days. For the CCB reinforced with Ca(OH)_2_-treated fibers, this increase reached 57% varying from 5.2 MPa after 7 days to 8.2 MPa after 90 days. In the unreinforced CCB, there was no significant difference during time, with a variation of only around 15% from 4.8 after 7 days up to 5.6 MPa after 90 days. Although the CCB reinforced with untreated fibers showed the highest variation, it did not reach the highest flexural value after 90 days. This was due, as mentioned before, to the fact that, during the first days, the water-soluble extractives in the fibers were released through the contact between untreated fibers and the alkaline medium of the cement, preventing the hydration of the cement core grains and, thus, retarding the cement setting, which resulted in a low flexural strength in the initial days. As the curing time increased, the fibers without extractives had more hydrogen active site to anchorage with cement grains, increasing significantly the flexural strength. Moreover, the roughness of the fibers after the Ca(OH)_2_ treatment, revealed by the SEM images, provided mechanical anchorage, which led to higher MOR values. It has been reported the improvement of the mechanical performance of CCBs with the inclusion of alkali-treated fibers. The values for MOR of these composites are related to the source of fiber and the used alkaline treatment. For bamboo fibers treated with sodium hydroxide, the most alkaline treatment used, CCBs shows flexural strength between 5 MPa to 12 MPa [37]. However, bamboo fibers treated with less environmental impact treatments, such as organosolv pulp, CCBs show maximum flexural stress of 8 MPa [38]. The mechanical results obtained with calcium hydroxide treatment demonstrate the potential of this treatment as an alternative to process *Guadaua* in cement-based composites.

The flexural strength exhibited a non-conventional behavior in relation to the W/C. A lower W/C commonly leads to a higher strength; however, the CCB reinforced with the fibers with highest W/C, showed also higher flexural strength. This trend could be due to the swelling capacity of the Ca(OH)_2_-treated fibers, which absorbed water during the mixing and successively released it during the curing, enhancing the water diffusion and the cement hydration, as reflected in the high flexural strength.

Upon analyzing the SE values, it was observed that the energy absorption was higher in the CCB reinforced with Ca(OH)_2_-treated fibers in the three curing stages; this might be related to the fiber surface modification by the alkali treatment, which resulted in less rigid fibers.

The Tukey test results for the MOR and SE after 7, 28, and 90 days of curing corresponded to three independent replicates per each mixture. After seven days, the MOR of the CCB reinforced with untreated fibers statistically differed (*p* < 0.05) from those of the other two samples; this could be due to the presence of extractives and non-cellulosic compounds in the untreated fibers, which delayed the cement hydration [39]. After 90 days, the MOR of both the reinforced CCBs were statistically different (*p* < 0.05) from that of the unreinforced one, indicating the positive effect of the reinforcement. On the other hand, at every curing stage, all the samples exhibited statistically different specific energies (*p* < 0.05) from each other.

The flexural results for the CCB reinforced with calcium Ca(OH)_2_-treated fibers showed a flexural strength suitable for fiber–cement flat sheets class 2 categories C and D, according to BS EN 12467:2012 [40], which are intended for internal applications, where they may be subjected to heat and moisture but not to frost.

### 3.6. Fracture Surface of CCBs

**Figure 11:**
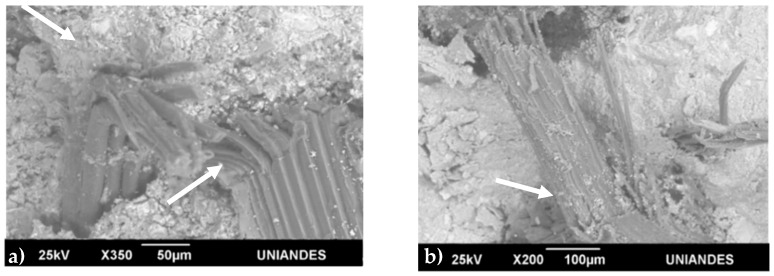

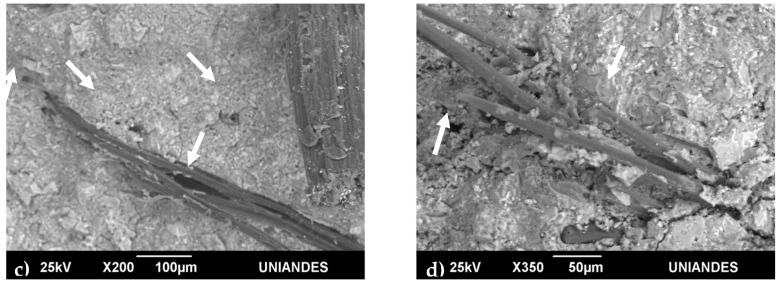
Scanning electron microscopy images of the fracture surfaces of composite cement boards reinforced with (**a**,**b**) untreated and (**c**,**d**) Ca(OH)_2_-treated *G. angustifolia* fibers, after (**a**,**c**) 7 and (**b**,**d**) 28 days.

After the flexural test, the fracture surfaces of the broken CCBs reinforced with fibers were observed by SEM to determine the main fiber failure mechanism (Figure 11). In the sample with untreated fibers, the fibers were fractured after the composite failure (Figure 11a,b); this mechanism is associated with a disruption of energy dissipation and weaker fibers, resulting in worse mechanical behavior. In the composite reinforced with Ca(OH)_2_-treated fibers, the surface modification by the alkali treatment provided fiber with better mechanical characteristics avoiding fracture. Thus, the fibers suffered pull out (Figure 11c,d) during the fracturing, allowing energy dissipation and, hence, endowing the CCB with a better mechanical performance. This fiber behavior into cement composite is reflected in the mechanical performance of CCB; where CCB reinforced with alkali-treated fibers showed the highest toughness and flexural strength. The performance on the fiber mechanical failure is attributed to the surface differences arising from the alkaline treatment [41].

The CCB reinforced with untreated fibers showed a prominent loss in bonding since the *G. angustifolia* fibers shrank strongly upon drying during curing.

Matrix cracking also occurred near the fibers, in both samples, because of the internal tensile stress generated by the fiber volume variation.

## 4. Conclusions

The calcium hydroxide treatment affected the surface and structure of all the *G. angustifolia* samples. It successfully removed the hemicelluloses and lignins from the fibers and disrupted the cell wall structure, increasing the cellulose crystallinity. Moreover, this alkali treatment was more efficient removing the non-cellulosic materials in the crushed sample compared with chips and barkless samples.

The mechanical properties of the CCB reinforced with Ca(OH)_2_-treated fibers, determined via modulus of rupture and toughness were higher than reinforced with untreated fibers and the unreinforced one (*p* < 0.05). This proves the efficiency of the Ca(OH)_2_ treatment of bamboo fibers for CCB production.

## Figures and Tables

**Table 1:** Characteristics of the ordinary Portland cement (type I), used in the composite.

Parameter	Value
Density (kg/m^3^)	2937
Specific surface (cm^2^/g)	4589.6
Unit mass (kg/m^3^)	825.9

**Table 2:** Cristallinity index (*CI*) of cellulose in the *Guadua* samples, before and after Ca(OH)_2_ treatment.

Sample	*I*_002_	*I*_am_	Cellulose *CI* (%)
Untreated	1557	829	46.8
*Guadua* chips	2419	1085	55.1
Barkless *Guadua*	1487	782.5	47.4
Crushed *Guadua*	2908	1009	65.3

**Table 3:** Water/cement ratios (W/C) of different composite cement boards (CCB).

CCB type	W/C
Without fibers	0.17 ± 0.01
With untreated fibers	0.23 ± 0.03
With Ca(OH)_2_-treated fibers	0.27 ± 0.01

**Table 4:** Average Modulus of Rupture (MOR) and Specific Energy (SE) of various composite cement boards (CCBs) after 7, 28, and 90 days of curing.

CCB Reinforcement	MOR (MPa)			Specific Energy (kJ/m^2^)		
	7 days	28 days	90 days	7 days	28 days	90 days
Unreinforced	4.8 ± 0.9 ^a^	5.2 ± 0.6 ^a^	5.6 ± 0.9 ^a^	0.19 ± 0.02 ^a^	0.20 ± 0.01 ^a^	0.25 ± 0.02 ^a^
Untreated fibers	3.6 ± 0.9 ^b^	5.0 ± 0.9 ^a^	7.4 ± 0.5 ^b^	0.63 ± 0.03 ^b^	0.51 ± 0.02 ^b^	0.61 ± 0.02 ^b^
Ca(OH)_2_-treated fibers	5.2 ± 0.8 ^a^	5.4 ± 0.6 ^a^	8.2 ± 0.9 ^b^	1.19 ± 0.20 ^c^	0.99 ± 0.09 ^c^	1.09 ± 0.10 ^c^

The same letter in the column means results do not differ by the Tukey Test (*p* < 0.05).
